# Actin-Binding Proteins as Potential Biomarkers for Chronic Inflammation-Induced Cancer Diagnosis and Therapy

**DOI:** 10.1155/2021/6692811

**Published:** 2021-06-05

**Authors:** Yu-Gui Zhang, Jiang-Tao Niu, Hong-Wei Wu, Xin-Lei Si, Shu-Juan Zhang, Dong-Hui Li, Tian-Tian Bian, Yue-Feng Li, Xing-Ke Yan

**Affiliations:** ^1^College of Pharmacy, Gansu University of Chinese Medicine, Lanzhou 730000, China; ^2^Key Laboratory of Standard and Quality of Chinese Medicine Research of Gansu, Engineering Research Center of Chinese Medicine Pharmaceutical Process of Gansu, Gansu University of Chinese Medicine, Lanzhou 730000, China; ^3^Laboratory of Molecular Biology, College of Acupuncture-Moxibustion and Tuina, Gansu University of Chinese Medicine, Lanzhou 730000, China

## Abstract

Actin-binding proteins (ABPs), by interacting with actin, regulate the polymerization, depolymerization, bundling, and cross-linking of actin filaments, directly or indirectly, thereby mediating the maintenance of cell morphology, cell movement, and many other biological functions. Consequently, these functions of ABPs help regulate cancer cell invasion and metastasis when cancer occurs. In recent years, a variety of ABPs have been found to be abnormally expressed in various cancers, indicating that the detection and interventions of unusual ABP expression to alter this are available for the treatment of cancer. The early stages of most cancer development involve long-term chronic inflammation or repeated stimulation. This is the case for breast cancer, gastric cancer, lung cancer, prostate cancer, liver cancer, esophageal cancer, pancreatic cancer, melanoma, and colorectal cancer. This article discusses the relationship between chronic inflammation and the above-mentioned cancers, emphatically introduces relevant research on the abnormal expression of ABPs in chronic inflammatory diseases, and reviews research on the expression of different ABPs in the above-mentioned cancers. Furthermore, there is a close relationship between ABP-induced inflammation and cancer. In simple terms, abnormal expression of ABPs contributes to the chronic inflammation developing into cancer. Finally, we provide our viewpoint regarding these unusual ABPs serving as potential biomarkers for chronic inflammation-induced cancer diagnosis and therapy, and interventions to reverse the abnormal expression of ABPs represent a potential approach to preventing or treating the corresponding cancers.

## 1. Introduction

Actin is an abundant and highly conserved protein that is mainly found in eukaryotic cells. The first myosin was extracted from frog muscle in 1887 [1], and then, the first actin was discovered during the 1940s in muscle and was later named actin due to its impact on myosin activity [2]. Researchers now have a relatively complete understanding of the structure and main functions of actin, including its mechanism of polymerization, the formation and structure of actin filaments and their complexes, and factors influencing the formation of actin filaments [3]. Actin in eukaryotic cells exists in two main forms: globular actin monomers (G-actin) and actin filaments (F-actin). F-actin is formed by the polymerization of G-actin, and the degree of actin polymerization (G-actin/F-actin ratio) is crucial in this process [4]. The actin cytoskeleton, as the primary force-generating machinery in the cell, is a collection of F-actin with accessory and regulatory proteins [5]. The dynamics of actin cytoskeleton is regulated by actin-binding proteins (ABPs) which participate in nucleation, elongation, and disassembling according to the need of the cells and play an important role in various biological systems, including muscle contraction, cytokinesis, cellular motility, plasmid separation in prokaryotes, and maintenance of cells and their physical integrities [6]. ABPs, a class of proteins that also serve as a bridge between the cell membrane and the nucleus, transmit signals within the cytoplasm from outside to inside and from inside to outside [7]. Since ABPs were first discovered in the 1970s, more than 160 different ABPs have been identified [8]. They are divided roughly into eight different types according to the differences resulting from virtually regulating every aspect of actin assembly as shown in Table 1 [9]. Collectively, these different types of ABPs have a variety of functions that enable a large number of actin molecules to polymerize, assemble into new filaments, promote elongation of these filaments, cap barbed or pointed ends to terminate elongation, sever filaments, and cross-link filaments [10].

The cytoskeleton not only participates in cellular motility and adhesion but also acts as a medium for signal transduction. Changes in type and number of cytoskeletal molecules are related to transfer-related phenotypes of tumors [11]. Moreover, ABPs had been implicated in cancer cell invasion and metastasis, chromosomal remodeling, transcriptional regulation, DNA damage repair, and protein-nucleocytoplasmic shuttle during various carcinogenetic processes [12, 13]. Therefore, biomarkers like this related to growth factors or tumor promoters/suppressors serve as targets for predicting cancer [14]. For instance, the ecotropic viral integration site 1 gene [15] and protocadherin17 promoter methylation [16] are two biomarkers to predict the prognosis of patients with clear cell renal cell carcinoma; the microRNAs (such as miR-122, miR-375, miR-92a, miR10a, and let-7c) are identified as biomarkers for acute or chronic HBV and HCV virus infection [17]; anoctamin-1 is a promising biomarker for esophageal cancer prognosis and precancerous lesion development prediction [18].

Studies have confirmed that most chronic inflammation diseases, including cancers, are caused by dysregulation of the inflammatory response at a molecular level [19]. Chronic inflammatory induction affects the sooner or later stages of tumor development [20, 21]. Anti-inflammatory therapies have been proposed to target the tumor microenvironment [22]. The immune system has a significant impact on regulating inflammation. Accordingly, approaches to treating cancer focus on natural genetic variations affecting inflammation and immunity [23, 24]. It was found that abnormal expression of ABPs occurs in related chronic inflammatory diseases, as shown in Table 2. Meanwhile, a variety of ABPs have been found to be abnormally expressed during metastasis, spread, or invasion of various cancers, including breast cancer, gastric cancer, lung cancer, prostate cancer, liver cancer, pancreatic cancer, esophageal cancer, and melanoma as shown in Table 3. Exploring the relationship among ABPs-inflammation-cancer, as a result, we think that abnormally expressed ABPs help the chronic inflammation develop into cancer and propose that these ABPs serve as potential diagnostic or therapeutical biomarkers for the chronic inflammation-induced cancer occurrence, development, progression, and prognosis. It is necessary to control the positive development of inflammation in order to prevent the occurrence of early cancers. What is more, the hope is to prevent long-term induced inflammation from developing into cancer by altering the abnormal expression of ABPs.

## 2. Chronic Inflammation Leads to Various Cancers

**Figure 1:**
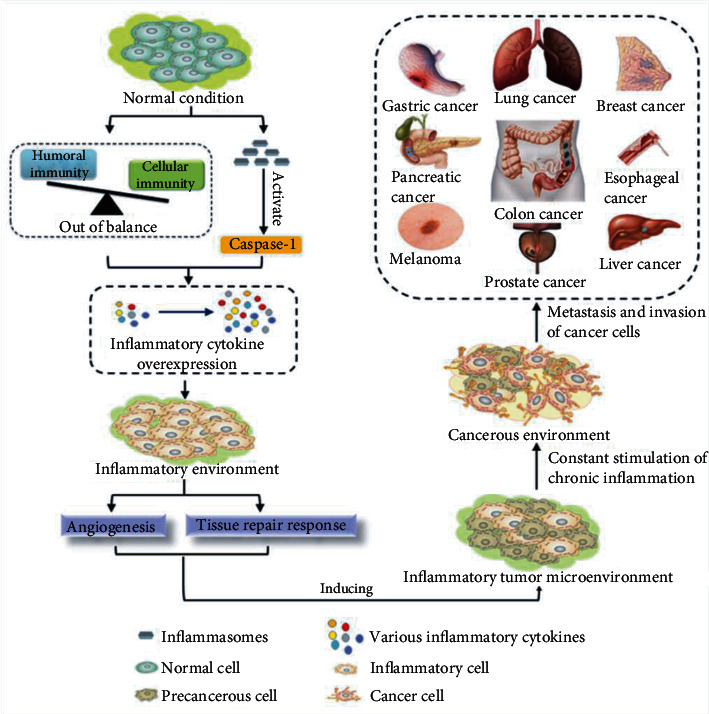
A process of chronic inflammation inducing various cancers. Inflammasomes are innate immune system receptors and sensors that recognize multiple microbial stress and injury signals, thereby directly activating caspase-1, inducing the secretion of proinflammatory cytokines, and ultimately leading to the occurrence of inflammation-related diseases. Humoral immunity and cellular immunity are out of balance, the overall expression of IC and Ig related to cellular immunity increases, and the Th1 response and Th2 response related to decent immunity show an overall downward trend, with increased expression of inflammatory cytokines such as IL-1, IFN-*γ* IL-4, IL-6, IL-10, and IL-13. Inflammation activates angiogenesis and the tissue repair response, induces the proliferation of precancerous cells and the formation of an inflammatory tumor microenvironment, and promotes the proliferation of precancerous cells, which evolve into cancer cells and then metastasize and spread, resulting in multitissue and organ carcinogenesis. IC: immune complex; Ig: immunoglobulin; Th: helper T cell; IL: interleukin; IFN-*γ*: interferon-*γ*. [24, 145–148].

The relationship between cancer and inflammation was first discovered in the 19th century. Leukocytes are found at tumor sites and also appear at sites of chronic inflammation. Meanwhile, “lymphoreticular infiltrate” reflected the origin of cancer at sites of chronic inflammation [25]. Inflammatory cytokines, such as TNF-*α*, IL-1*β*, and IL-6, are also found in tumor biopsy samples. They suppress the immune system, promote tumor growth, and then regulate tumor development in different directions [25, 26]. In addition, macrophages are the main immune infiltrating cells in tumors and are the key cell types connecting inflammation and cancer. The most direct association between inflammation and cancer arises from damage to organ DNA, which leads to mutated DNA and induces cancer [27]. This is one of the most common molecular mechanisms in cancer. Although the mechanism by which inflammation promotes cancer is not fully understood, two interrelated hypotheses have emerged. One involves an internal pathway driven by genetic changes that cause tumors and inflammation. The other is that inflammatory conditions increase cancer risk via an external process [28]. Inflammation-induced tumor promotion occurs in all stages of tumor development and leads to the activation of precancerous lesions that have been latent for many years. On the contrary, inflammation in the tumor microenvironment promotes the proliferation and survival of malignant cells, angiogenesis, metastasis, destruction of adaptive immunity, and responses to hormones and chemotherapy drugs [25, 29, 30] The above-mentioned reasons hinder the successful treatment of cancers. The fact that chronic inflammation (or inflammation) leads to various cancers is shown in Figure 1 and the paragraph below.

Gastritis is caused by long-term infection with *Helicobacter pylori* (HP). Long-term chronic gastritis stimulation is the key to the activation of gastric cancer cells [31]. The overexpression of gastrointestinal inflammatory cytokines such as IL-6 and IL-17 increases the severity of the tumor. Tumor formation will lead to an imbalance of intestinal flora and inflammation [32]. The balance of the intestinal microenvironment directly affects the occurrence and transformation of carcinogenic structures [33]. Moreover, colitis promotes the development of tumors by changing the microbial composition [34], which ultimately leads to colitis-associated cancer. Therefore, the consolidation of intestinal inflammation is the key to preventing colitis from developing into colon cancer. Recent research has shown that upregulation of intracellular proinflammatory factors (such as IL-1*β*, IL-6), *transforming growth factor-β* (TGF-*β*) and DNA hypermethylation inhibit the expression of miR-34b-5p, activate CRL4^DCAF4^ E3 ligase, ubiquitinate the suppression of tumorigenicity 7, and result in its degradation, thereby mediating the colitis-associated tumorigenesis [35]. The occurrence of esophageal cancer is also related to esophageal squamous epithelium chronic inflammation. This condition causes DNA damage and alters the expression of genes involved in cellular proliferation and apoptotic inhibition, resulting in the occurrence of esophageal tumors [36, 37]. This response is similar to the transition from gastritis to gastric cancer, with the presence of a tumor-infiltrating pattern of immune cells under inflammatory infiltration [38]. Primary liver cancer involving malignant tumors is another typical example of chronic inflammation-related cancer. More than 90% of liver cancers are associated with chronic liver injury and liver inflammation. Mechanisms of inflammation turning into hepatocarcinogenesis mainly involve activation of signaling pathways (like NF-*κ*B pathway, JAK-STAT signaling, epidermal growth factor receptor signaling, and epidermal growth factor receptor signaling), cytokines (like IL-1*α*, IL-1*β*, IL-6, IL-8, and TNF-*α*), oxidative DNA damage, DNA methylation, and hepatocyte injury [39]. Continuous stimulation of the inflammation-related IL-6 pathway contributes greatly to the activation of hepatocellular carcinoma, which eventually leads to the proliferation and invasion of liver tumors [40]. The cytokines like TNF-*α* and IL-6 and their downstream targets *nuclear factor κB* (NF-*κ*B) c-Jun N-terminal kinase and STAT3 drive inflammation-related hepatocellular carcinoma. Adaptive immune cells like CD8^+^ T cells, Th17 cells, and B cells also stimulate the development of liver cancer [41]. The inflammatory cytokine TNF-*α* also stimulates tumor progression in *non-small-cell lung cancer* (NSCLC). The expression level of TNF-*α*-inducible protein 2 (TNF-*α*-IP2) is significantly increased in tumor tissues. Silencing TNF-*α*-IP2 reduces lung cancer cells' survival rates and inhibits their invasion and migration [42]. Breast cancer is closely related to chronic inflammation, especially *white adipose tissue* (WAT) inflammation in the breast [43, 44]. A study has shown that miRNAs (like miR-30, miR-146, and miR-205) associated with inflammation are used as potential biomarkers and therapeutic targets for breast cancer [45]. *Toll-like receptors* (TLRs) are activated by microbe-associated molecular patterns (MAMPs), which activate inflammatory pathways, including NF-*κ*B [46]. This increases levels of several proinflammatory cytokines (like TNF, IL-1, and IL-6) and the secretion of WAT ligands. Exacerbated inflammation aids in breast tumor progression and metastasis [47, 48]. This mechanism greatly increases the risk of breast cancer. One of the major causes of pancreatic cancer is the long-term persistence of pancreatitis. Pancreatitis promotes cancer-associated inflammation through activation of immune receptors such as macrophage and bone marrow dendritic cells, leading to carcinogenesis [49]. The infiltration of inflammatory cells during this process contributes to the growth and metastasis of the tumor. In particular, TNFs have a key role in increasing the risk of cancer, promoting cancer growth, and leading to cancer-related cachexia [50]. Furthermore, targeted inhibition of IL-6 enhances the efficacy of the antiprogrammed death-1-ligand 1 in pancreatic ductal adenocarcinoma to express antitumor activity [51]. Proliferative inflammatory atrophy and prostatic intraepithelial neoplasia occur before prostate cancer. Heterogeneous signals from infiltrating immune cells result in cytokine addiction of cancer cells, including cancer stem cells [52, 53]. Besides, the prostate infection causes a breakdown of the epithelial barrier, leading to inflammatory changes such as proliferative inflammatory atrophy in the prostate microenvironment, which in turn contribute to the reprogramming of prostate epithelial cells to induce the initiation of prostate tumors [54]. Chronic skin or mucosal inflammation is a classic cause of melanoma [55]. Long-term exposure of the skin to intermittent intense ultraviolet light induces a neutrophilic inflammatory response. The resulting chronic skin inflammation helps to release a large number of inflammatory factors, such as TNF. TNF promotes melanoma cells to migrate towards and spread along blood vessel endothelial cell surfaces [56]. B cells are exposed to the secretory layer of melanoma in vitro and differentiate into plasma sheath-like cells [57]. With the expression of plasma sheath proteins in these cells, T cells recruit chemokines CCL3, CCL4, and CCL5 and increase the expression levels of cytokines IL-12A, IFN-*γ*, and IL-10 and chemokines CXCL9 and CXCL10 [57, 58]. These effects stimulate new angiogenesis and then promote the migration of melanoma cells to endothelial tissue and the formation and development of tumors in the skin and mucous membranes.

In short, there are strong correlations between chronic inflammation and various cancers as mentioned above. Compounds in the inflammatory tumor microenvironment include leukocytes, cytokines, and complement components (like C3, C5, and C5AR1 [59]), which are regulated by transcription factors (like NF-*κ*B and STAT3 [30]). Proinflammatory factors related to the tumor microenvironment include IL-6, IL-8, TNF-*α*, TNF-*β*, and FASL; the main relevant cytokines are IL-1, IL-2, IL-12, and IFN-*γ* [60, 61]. Immune cells such as CD8^+^ T cells, Th17 cells, and B cells are also involved. In the process of canceration, tumor cells are surrounded by stromal cells and immune cells, which promote the development of the tumor [62]. During this process, levels of proinflammatory cytokines and immune cells can be changed. Thus, these represent potential biomarkers for the diagnosis and treatment of the corresponding inflammatory diseases and cancers.

## 3. ABPs in Chronic Inflammatory Diseases

Inflammatory caspases such as human caspase-4 can regulate cell migration through actin cytoskeleton polymerization or remodeling, which helps halt intracellular replication of pathogens and reduces inflammation [63]. ABPs help regulate the actin skeleton polymerization. This modulation can activate inflammation-related NF-*κ*B signaling pathways and subsequently upregulate the expression of IL-6 and IL-8 in inflammatory diseases, such as periodontal ligament tissue inflammation [64]. For example, Arp2/3 complex-dependent actin skeleton remodeling is important for B cell activation to alleviate inflammatory responses [65]. Accordingly, ABPs contribute to chronic inflammation diseases. Some examples are shown in Table 2.

The vitamin *D-binding protein* (DBP) is an extracellular scavenger for actin released from damaged/dead cells. Whatever the inflammation is, DBP binds to G-actin to form DBP-actin complexes which respond to tissue injury immediately [66]. The functions of DBP involve transport of vitamin D metabolites, control of bone development, and binding of fatty acids in modulating inflammatory response [67]. Gelsolin has two different modes of action in the immune system. Extracellular gelsolin is involved in the recognition of bacterial wall molecules and the attack of immune components, such as macrophage and Rac1 protein. Intracellular gelsolin is important for the recruitment and movement of macrophages [68]. Gelsolin involves the immune process and interacts with different cells of the immune system, such as helping macrophages migrate, binding with neutrophils, inducing T lymphocytes, and driving abnormal expression of IL-6, IL-1*β*, and IL-9 at a normal level, making it a potential candidate for multiple therapeutic applications like cancer, Alzheimer's disease, and arthritis [69]. What is more, gelsolin regulates the actin assembly and disassembly in *psoriatic arthritis* (PsA). Thus, the expression of gelsolin reduces significantly in this kind of chronic inflammation [70]. *Thymosin beta 4* (T*β*4) is an actin-sequestering protein. It not only prevents pathological changes in lipopolysaccharide-induced acute liver injury mice but also blocks protein phosphorylation to inhibit NF-*κ*B activation and prevent the production of proinflammatory cytokines [71]. On the contrary, the inflammatory NF-*κ*B signaling pathway is activated by Tir-Nck and Tir-EspFu actin polymerization, which aggravates enteropathogenic *Escherichia coli*- (EPEC-) induced inflammatory responses in epithelial cells [72]. A study about infants born prematurely with *bronchopulmonary dysplasia* (BPD) found that highly expressed T*β*4 and profilin-1 are detected in lung epithelial cells. By function, these proteins are associated with inflammation in lung injury in immature infants [73]. Another study showed that ABP-280 (filamin) binds *stress-activated protein kinase* (SAPK) activator SEK-1, which is the ABP necessary for activating inflammatory TNF-SAPK in melanoma cells [74]. Introducing the protein into human melanoma cells was shown to inhibit TNF, IL-1, TLRs, and TNF *receptor-associated factor* 2- (TRAF2-) induced NF-*κ*B activation, thereby restoring the SAPK or NF-*κ*B response to TNF activation [75]. These experiments suggest the role of filamin in the inflammatory signal transduction pathway of tumor cells [76]. It has also been reported that changes in villin-1 and gelsolin alter the cytoskeleton in intestinal epithelial cells, inducing gastrointestinal inflammation [77]. The actin-binding protein *synaptopodin* (SYNPO) also regulates intestinal mucosal susceptibility and permeability and then achieves homeostatic balance in the intestinal tract of inflammatory bowel diseases [78]. Cortactin plays an important role in regulating actomyosin contractility and leukocyte transendothelial migration (diapedesis) in a cytokine-induced inflammation model in the cremaster muscle [79]. Cortactin also contributes to neural inflammatory development by supporting leukocyte transmigration through the blood-brain barrier [80]. These studies suggest that ABPs including gelsolin, T*β*4, filamin, villin-1, gelsolin, profilin-1, SYNPO, and cortactin have important roles in inflammation-related diseases.

## 4. Different Cancers and Related ABPs

Cancer remains difficult to treat because infiltrating tumor cells can migrate, enter the lymphatic circulation, and survive in multiple body parts [25, 81]. ABPs change the dynamic structure of actin and regulate tumor invasion and metastasis [12]. A large number of studies have shown that the content of ABPs that constitute the cytoskeleton increases or decreases during the migration of cancer cells. These changes in protein content are available for potential biomarkers in tumor diagnosis.

### 4.1. Breast Cancer

Metastatic breast cancer is driven by deep remodeling of the cytoskeleton, which allows tumor cells to effectively migrate and invade [82]. To date, ABPs that have been implicated in breast cancer cell migration, invasion, and growth include *α-actinin 4 (ACTN4)*, *actin filament-associated protein* (AFAP-110), *coronin-like actin-binding protein 1C* (CORO1C), girdin, and *anillin* (ANLN). ACTN4 is a member of the *α*-actinin family of actin cross-linking proteins. Upregulated ACTN4 plays a specific role in the metastasis of cancer. ACTN4 is also a part of the cellular reflex system. This role allows tumor cells to mount a more specific response such as calcium signaling, PIP3 synthesis, or downstream of chemokine signaling [83, 84]. Knockout of the ACTN4 gene significantly reduced the expression of estrogen receptor-*α* in MCF-7 breast cancer cells [85]. AFAP-110 is an adaptor protein that modulates changes in actin filament integrity. AFAP-110 is not only expressed in normal muscle epithelial cells but also highly expressed in human breast cancer MDA-MB-231 cells [86]. MDA-MB-231 cells require AFAP-110 expression to form stress fibers and for adhesion, indicating an important role of AFAP-110 in breast cancer cell adhesion [87]. In addition, the migration and invasion of MDA-MB-231 cells are suppressed by silencing CORO1C, a downstream target of Y-box binding protein-1, which is a ribosome-binding protein that maintains the homeostasis of epidermal progenitor cells [88]. Girdin, an Akt substrate that binds to actin, is also expressed in MDA-MB-231 cells and assists in the process of MDA-MB-231 cell migration, indicating the important role of girdin in tumor progression in which the Akt signaling pathway is aberrantly activated [89]. An ABP ezrin phosphorylation at the carboxyl terminal *threonine 567* (Thr567) is enhanced by *17b-estradiol* (E2). The action involves estrogen receptor interaction with the nonreceptor tyrosine kinase c-Src, which activates the small GTPase *RhoA/Rho-associated kinase* (ROCK-2) complex and the phosphatidylinositol-3 kinase/Akt pathway, and finally promotes breast cancer cell movement and invasion [90]. These results demonstrate that estrogen and ezrin have positive induction effects on breast cancer. It has also been reported that breast cancer patients with high expression of ANLN have a poor prognosis [91]. Knockout of ANLN leads to suppression of stemness and induction of mesenchymal-to-epithelial transdifferentiation, indicating that inhibiting ANLN expression hinders breast cancer cell migration and invasion [92].

### 4.2. Gastric Cancer

Gastric cancer is closely related to HP [93]. HP damages the gastric mucosa, changes the release pattern of gastric hormones, affects gastric physiology, and causes chronic gastritis and peptic ulcers. This is a long-term process that ultimately leads to gastric cancer [31]. The process by which HP-induced gastritis develops into gastric cancer involves multiple molecular mechanisms, including contributions of ABPs. The fascin protein has a supporting role in the spread of gastric tumors. Studies have shown that IL-6 upregulates fascin expression levels in MKN45 gastric cancer cells, thereby promoting *signal transduction and activator of transcription 3*- (STAT3-) dependent migration and invasion. STAT3 directly regulates the expression of the fascin protein. NF-*κ*B binds to the fascin promoter in a STAT3-dependent manner. Thus, the STAT3-NF-*κ*B-fascin signaling axis was identified as a therapeutic target to block the invasion and migration of gastric cancer cells [94]. Fascin is also an important prognostic factor in gastric cancer. Knocking out fascin-1 was shown to inhibit the migration of gastric cancer cells [95]. Besides, overexpression of ANLN is a molecular predictor of fortestinal- and proliferative-type gastric tumors. Specifically speaking, ANLN has a significant positive association with Wnt/*β*-catenin signaling and a negative association with ER-*α*signaling [96]. Furthermore, an ABP *capping actin protein*, *muscle Z-line alpha subunit 1* (CAPZA1), is overexpressed in gastric epithelial cells infected with HP [97]. Knocking out CORO1C significantly reduced the total number of gastric cancer cells, inhibited cell viability, cell colony formation, mitosis, and metastasis, and promoted apoptosis [98]. *Yes-associated protein* (YAP) causes cytoskeletal rearrangement by changing the dynamics of the F-actin/G-actin turnover, thereby promoting the migration of gastric cancer cells [99]. The above results indicate that overexpression of ABPs such as fascin, ANLN, YAP, CORO1C, and CAPZA1 promotes gastric cancer cell activity.

### 4.3. Lung Cancer

Lung cancer is the leading cause of cancer deaths in male patients worldwide [100]. Surveys have shown that smoking, age, radon exposure, environmental pollution, occupational exposure, gender, race, and preexisting lung disease are important factors in lung cancer [101]. Research related to ABPs and lung cancer has mainly focused on *twinfilin-1* (TWF1) and fascin-1 and on the relationships between VASP, profilin-1, and cofilin-1. VASP, cofilin-1, and profilin-1 regulate cell proliferation and migration by regulating the dynamics of actin [102, 103]. Studies have examined lower profilin-1 and elevated cofilin-1 levels observed in infant *bronchopulmonary dysplasia* (BPD) tissue. The reason is related to decreasing availability of actin monomers and increasing actin endcapping, which subsequently leads to an impaired tissue or cellular repair mechanism [104]. Another study confirmed that TWF1 mRNA has a binding site for miR-486-5p in the 3′ untranslated region. Expression levels between TWF1 and miR-486-5p are the negative correlations. As a consequence, the role of TWF1 in promoting cisplatin resistance in NSCLC is regulated by miR-486-5p [105]. A recent study found that expression levels of profilin, fascin, and ezrin mRNA in patients with NSCLC lymphocyte metastasis are significantly increased [106], indicating that increased levels of these ABPs connected with metastasis or invasion of lung cancer cells are used as predictors of lung cancer. ANLN was shown to undergo genetic changes and overexpression at the RNA and protein levels in patients with lung cancer; this is associated with poor prognosis [100]. Fascin-like proteins also have a role in lung cancer. In NSCLC, fascin-1 is a direct target of miR-145, which has an inhibitory effect on the migration and invasion of NSCLC cells [107]. Therefore, the expression level of fascin-1 is used to evaluate prognosis in NSCLC patients.

### 4.4. Prostate Cancer

The main current treatments of prostate cancer are surgical resection, radiotherapy, and hormone therapy [108]. In recent years, immunotherapy, especially adoptive immunotherapy, has shown great benefits for patients with advanced prostate cancer. Recent research showed that plasma let-7f-5p combined with a prostate-specific antigen is used as a biomarker for the diagnosis of prostate cancer [109, 110]. The F-actin-binding protein *switch-associated protein* 70 (SWAP70, SWAP switch B cell complex 70 kDa subunit) is involved in the activation of B cell transformation. A study of 75 clinical prostate specimens using SWAP70 immunohistochemical analysis showed that silencing SWAP70 significantly inhibited migration and invasion of prostate cancer cell lines. These results show that SWAP70 has a potential carcinogenic function and indicate new molecular mechanism-based approaches for the treatment of prostate cancer [111]. Another study showed that *PDZ and LIM domain protein 4* (PDLIM4) is a potential molecular marker. PDLIM4 binds directly with F-actin, which regulates cytoskeletal function. The expression of PDLIM4 mRNA and protein in human prostate tumorigenic PC3 cells is roughly half of that in nontumorigenic RWPE1 cells. In addition, the reexpression of PDLIM4 inhibits prostate cancer cell growth, proliferation, and clonogenicity [112]. In a recent study, a long noncoding RNA associated with lung adenocarcinoma transcript 1 (MALAT1) is isolated from CORO1C; silencing of MALAT1 was shown to inhibit the migration, invasion, and epithelial-mesenchymal transition of prostate cancer cells [113].

### 4.5. Liver Cancer

Liver cancers include hepatocellular carcinoma, intrahepatic cholangiocarcinoma, and other rare tumors, such as fibrous lamellar carcinoma and hepatoblastoma [114]. Hepatocellular carcinoma is one of the most common and invasive cancers. Long-term alcohol misuse and viral hepatitis lead to chronic liver damage, which are promoters of liver cancer [115]. ANLN is an evolutionarily conserved ABP. The number of ANLN genes in liver cancer cells affects cell division. Knockdown of ANLN in H2.35 hepatocytes using a small interfering RNA resulted in increased expression levels of ANLN mRNA in human liver cancer tissues; furthermore, the cytoplasmic division was blocked, which inhibited the development of liver tumors. These results indicate that drugs inhibiting ANLN in the liver are effective in the prevention or treatment of liver cancer [116]. Another study found high levels of the actin-bound 50 kDa protein in rat liver tumors [117]. Therefore, the 50 kDa protein is a candidate for the interaction between actin and the plasma membrane of hepatocytes and has positive significance in the treatment of liver tumors.

### 4.6. Pancreatic Cancer

Uncontrolled cell division and growth in the pancreas contribute to pancreatic cancer formation and development. Its risk factors include smoking, chronic pancreatitis, obesity, long-term diabetes, a family history of pancreatic cancer, and high consumption of red and processed meat [118]. In recent years, the number of pancreatic cancer patients has risen sharply, and it has become the seventh leading cause of cancer deaths [119]. Therefore, there is an urgent need to elucidate the biological mechanisms of pancreatic cancer and identify biomarkers for its diagnosis and treatment [120]. The function of the ABP destrin has been reported to be affected by LAMC2/NHE1 signaling, and its receptors are highly expressed in pancreatic cancer tissues and cells. LAMC2 phosphorylates Akt-Ser473 to promote NHE1 expression, activity, and cell membrane accumulation in pancreatic cancer cells. Activating Akt/NHE1 signaling to mediate the invasion of pancreatic cancer cells was shown to upregulate the expression of destrin in pancreatic cancer cells. Destrin is upregulated in nerve-infiltrating pancreatic cancer cells, and its expression is related to invasiveness around nerves [121]. Moreover, cortactin and fascin-1 are overexpressed in the tissues of patients with advanced pancreatic cancer, and this is associated with low rates of long-term survival [122]. Thus, blockade of destrin, cortactin, and fascin-1 overexpression will slow down pancreatic cancer progression.

### 4.7. Esophageal Cancer

The two main histological types of esophageal cancer are squamous cell carcinoma and adenocarcinoma [123]. A study showed that natural macrolide F806 inhibits the dynamic assembly of F-actin *in vitro*, thereby inhibiting the invasion and metastasis of esophageal squamous cell carcinoma (ESCC) cells. This is related to F806 preventing the aggregation of the paxillin protein, which forms focal adhesions by binding to the ends of actin filaments [124]. Previous studies have shown the combined protein expression pattern of four ABPs, tensin, profilin-1, villin-1, and talin, as biomarkers to assess the prognosis of ESCC patients [125]. Therefore, immunohistochemistry is used to detect the expression of profilin-2 protein on ESCC tissue chips for the Han and Kazakh ethnic groups. The expression of profilin-2 in intraepithelial neoplasia and ESCC is significantly increased. Downregulation of profilin-2 inhibits the ESCC cells' invasion and migration, as well as inducing an EMT phenotype. Hence, profilin-2 represents a promising biomarker for ESCC treatment [126]. The actin-bound Akt substrate girdin is involved in the motility of ESCC cells, and its expression levels are inversely related to the survival of ESCC patients. Therefore, girdin is a prognostic marker for ESCC [127]. Another study showed that the expression of fascin is involved in cytoskeletal alterations such as reducing expression of b-catenin and c-erbB-2. Fascin also participates in cell protrusions and proliferation formation and promotes invasiveness and metastasis in carcinogenesis. Therefore, fascin plays a crucial role in regulating the neoplasm progression of ESCC [128].

### 4.8. Melanoma

Most melanomas originate in the skin, but they also occur in the mucous membranes of the respiratory tract, digestive tract, and reproductive tract [129]. The current understanding of melanoma includes the following main characteristics: self-sufficiency of growth factors, insensitivity to growth inhibitors, escape of apoptosis, unlimited replication potential, continuous angiogenesis, tissue invasion, and metastasis [130]. The main treatment for melanoma is immunotherapy; thus, the search for biomarkers for use in the treatment of melanoma on the basis of biology is a research focus [130, 131]. Espin plays an important role in melanoma cell metastasis. The expression of espin in melanoma mice is significantly increased by immunohistochemistry. Knockdown of espin leads to significantly less metastasis of melanoma cells [132]. Furthermore, espin is targeted by miR-612 to inhibit the invasive phenotype of melanoma cells [133]. Thus, espin represents a new biomarker for melanoma progression. Melanoma cells also exhibit abnormal localization of ABP cortactin, which corresponds to the colocalization of filamentous actin in the cultured melanoma cell cortex. It was also found that among the 170 melanocyte lesions (including 106 cutaneous *malignant melanoma* (MM), 24 *dysplastic nevi* (DN), and 40 *common melanocytic nevi* (CMN)) collected, cortactin is strongly positively expressed in all three types of melanoma. But there is no statistical difference in cortactin immunostaining intensity among CMN, DN, and MM [134, 135], indicating that the protein is highly expressed in different types of melanomas. This implies a potential role of cortactin in the regulation of melanoma progression and provides a reasonable basis for targeted intervention in melanoma treatment.

### 4.9. Others

In addition to the cancers mentioned above, ABPs are abnormally expressed in rectal cancer, basal cell carcinoma, and trophoblastoma. Transgelin is a 23 kDa ABP and a candidate biomarker for lymph node status [136]. Its expression is significantly increased in colorectal cancer [137]. Drebrin expresses at medium and high levels in the lysate of squamous cell carcinoma cell line DJM-1 and that of the normal human epidermis. Drebrin is also detected at the cell-cell junction in normal human epidermal tissue, and its expression is mainly concentrated at the tumor cell-cell boundary. This indicates that drebrin has significance in the differential diagnosis of basal cell carcinoma, glioblastoma, and hair epithelioma [138].

Our above discussion has shown that the expression level of most ABPs is positively correlated with proliferation and invasion of related cancer cells, except TWF1, cofilin-1, and PDLIM4. Furthermore, the coexpression of multiple ABPs in certain cancer is persuasive in judging the development or prognosis of cancer: for example, the overexpression of both cortactin and fasin-1 in the tissues of patients with advanced pancreatic cancer [122], the expression patterns of tensin, profilin-1, villin-1, and talin collective protein available as biomarkers to evaluate the prognosis of ESCC patients [125], and the coexpression of VASP phosphorylation, profilin-1, and cofilin-1 in lung cancer tissues as biomarkers for lung cancer development [104]. Few studies have focused on the coexpression of multiple ABPs in cancer, which, on the contrary, needs more attention from scientists.

In terms of different types of ABPs, our discussion shows that actin monomer-binding proteins (such as profilin family, twinfilin, and transgelin) are mainly related to lung cancer and breast cancer; actin filament polymerases (VASP) are related to lung cancer; severing proteins (cofilin-1) are related to lung cancer and pancreatic cancer. The overall expression trend of similar proteins is negatively correlated with the proliferation of cancer cells; the overall expression trend of severing proteins is negatively correlated with the proliferation of cancer cells; nucleation proteins (ARP2/3) and capping proteins (ARP2/3 and CAPZA1) are related to colorectal cancer and lung cancer, especially gastric cancer; cross-linking proteins (fimbrin and ACTN4) are closely linked to breast cancer and melanoma; filament-binding proteins (AFAP-110 and girdin) are mainly related to breast cancer, gastric cancer, lung cancer, and prostate cancer; and bundling proteins (such as fascin, fimbrin, and villin) are related to lung cancer, prostate cancer, and melanoma, especially prostate and esophageal cancers. Most ABPs are abnormally expressed in lung cancer, followed by breast and gastric cancers. Consequently, the systematic research on different types of ABPs and corresponding cancers will have more practical significance in the future. The steady-state problem in a pathological state of organism [139] for the abnormal expression of certain ABPs or the coexpression of multiple actins or different types of actins needs to be considered. However, this is not the main topic of this paper and will not be addressed here.

The abnormal expression of a variety of ABPs in cancer is listed in Table 3; nevertheless, this paper only analyzes the correlation between different types of ABPs and cancer on the basis of existing data due to the diversity of ABP functions. This means that how to clearly and specifically classify ABPs will not be included in our article.

## 5. Abnormally Expressed ABPs as Promoters of Chronic Inflammation into Cancer

As discussed above, various ABPs belong to abnormal expressions in cancers. Cancers are induced by chronic inflammation, and several ABPs are involved in the pathogenicity of chronic inflammatory diseases. However, how is the expression of associated ABPs during the whole process of chronic inflammation-induced cancers? Whether the abnormal expression of ABPs occurs only during inflammation or after cancer? These are thought-provoking issues.

**Figure 2:**
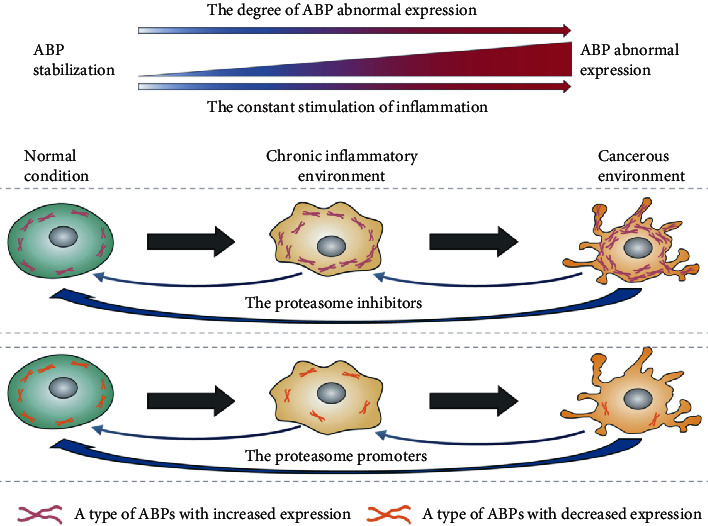
Abnormally expressed ABPs as promoters of chronic inflammation into cancer. ABPs have been already abnormally expressed in the early stage of chronic inflammation, and the degree of ABP abnormal expression increases with the continuous stimulation of inflammation and the occurrence of cancer. If the proteasome inhibitors for the overexpression of some ABPs and the proteasome promoters for the underexpression of other ABPs can be found in the cancer environment, this will help reverse the abnormally expressed ABPs in the cancerous environment to achieve stabilization, which will have an important impact on the treatment of chronic inflammation-induced cancer. For example, the proteasome inhibitor miR-146a regulates the overexpression of fascin in chronic inflammation-related colon carcinogenesis to achieve stabilization [142]. However, further research is needed on the expressions of remaining ABPs.

Based on the preceding context, we assume that the inflammation at the outset is accompanied by abnormal expression of ABPs. The degree of abnormal expression of ABPs will increase with the aggravation of chronic inflammation, which then helps cancer cells proliferate, metastasize, and invade, as shown in Figure 2. This assumption has been proven by other studies. The actin-bundling protein fascin is overexpressed in *inflammatory bowel disease* (IBD), and *nitric oxide* (NO) derived from chronic inflammation is a candidate for fascin upregulation [140, 141]. Some studies use the LC-MS/MS technique to find that fascin, as one of the nine highly expressed actin-related cytoskeleton proteins, has an elevated expression in *adenocarcinoma cells derived from FPCK-1-1 cells in the chronic inflammation* (FPCKpP-3) converted from *human colonic adenoma cells* (FPCK-1–1) by chronic inflammation. According to western blot analysis, fascin is highly expressed in all FPCKpP cell lines but low in FPCK-1-1 cell lines. Upregulation of fascin is generally observed in different bowel carcinogenesis accelerated by chronic inflammation. The tumorigenic potential of colon tumor cells is regulated by fascin expression *in vivo*. Fascin modulates caspase-3-dependent apoptosis cascade inhibiting anoikis and regulates colon carcinogenesis. The analysis demonstrated that fascin is an accelerator of the conversion of colonic adenoma cells into adenocarcinoma cells by chronic inflammation [140]. Another study shows that fascin is a key regulator of FPCKpP1-4 tumorigenicity and is overexpressed as the result of the suppression of proteasomal degradation accompanied by inflammation-induced miR-146a [142]. miR-146a is identified as a biomarker due to overexpression in the colonic mucosal epithelium of IBD patients [143] which inhibits proteasomal degradation [144]. Treatment with the proteasome inhibitor also restores fascin normal expression. This thus suggests that fascin accumulation, caused by reduced proteasomal activity, contributes to the acquisition of cancer stemness in chronic inflammation-related colon carcinogenesis [142]. However, further research is needed on the expressions of remaining ABPs in corresponding chronic inflammation-related cancers.

## 6. Conclusion and Prospects

Based on the above, chronic inflammation promotes the occurrence and development of cancer, and it participates in various pathological processes during cancer occurrence, growth, and metastasis. Breast cancer, gastric cancer, lung cancer, prostate cancer, liver cancer, esophageal cancer, pancreatic cancer, melanoma, colon cancer, etc. are related to early chronic inflammation, as has been described in detail above. Owing to the effects of continuous inflammation, the tumor microenvironment will gradually form, and large amounts of proinflammatory factors, cytokines, etc. will be released into this environment to promote the formation, development, and spread of tumor cells. ABPs such as gelsolin, T*β*4, filamin, villin-1, gelsolin, profilin-1, SYNPO, and cortactin are abnormally expressed in certain inflammatory environments (Table 2), where they inhibit or induce immunodeficiency inflammation.

ABPs are an important focus of cancer research owing to their ability to change the structure of actin, maintain cell morphology, cell movement, and many other biological functions, and affect the occurrence and development of tumors. Many ABPs are potential biomarkers for cancer diagnosis and therapy. What is more, different types of ABPs should have different focuses on cancer types, and the common abnormal expression of multiple ABPs in a certain cancer is also an important reason for accelerating cancer progression. This discovery provides new ideas for the research of cancer with various functions of ABPs and suggests that future researches should focus on the coexpression of multiple ABPs in certain cancer, rather than a single ABP study. It has more practical significance for discovering biomarkers in cancer.

Besides, the abnormally expressed ABPs are the accelerant of chronic inflammation into cancer. To date, 160 different ABPs have been identified; however, few studies have examined the abnormal expression of ABPs in chronic inflammation and chronic inflammation-induced cancer. Thus, further study should focus on the expression of different ABPs in the major types of chronic inflammation, as well as the specific ABPs expressed during the process of inflammation-induced cancer development. Furthermore, which ABPs are used as biomarkers needs to be intensively studied. It is hoped that such research will elucidate the mechanisms involving these ABPs and offer means to prevent or treat the occurrence and development of cancer by regulating their expression.

In conclusion, ABPs are considered candidate diagnostic and therapeutic biomarkers for cancers involving chronic inflammation. Further and more comprehensive studies should be conducted in the future. For most ABPs, research in this area has been insufficient. Therefore, many studies will be required to expand the literature and provide an experimental basis for the corresponding clinical methods.

## Figures and Tables

**Table 1:** Eight different types of ABPs and their family members.

Types	Members	Ref.
Actin monomer-binding proteins	Profilin family, vitamin D binding protein, anillin, transgelin, twinfilin, thymosin-*β*4, WASp-homology 2, cofilin family, etc.	[3, 149]
Severing proteins	Cofilin family, gelsolin family, destrin, villin, depactin, actophorin, etc.	[3, 121]
Nucleation proteins	Arp2/3 complex, formins, spire, cordon bleu, leiomodin, etc.	[3, 150, 151]
Actin filament polymerases	ENA/vasodilator-stimulated phosphoprotein, profifilin, mDia1, etc.	[3, 152]
Capping proteins	Tropomodulin, heterodimeric capping protein, Arp2/3 complex, barbed-end capping proteins, fragmin, severin, gelsolin, villin, V-1/myotrophin, sequesters, gelsolin family, coronin-like 1C, muscle Z-line alpha subunit 1, etc.	[3, 71, 97, 149, 153–156]
Cross-linking proteins	Fimbrin, *α*-actinin family, fodrin, actinogelin, caldesmon, gelactin family, mapping filamin, fascin, actin-binding domains, EF hand proteins, calcium-binding motif, paxillin, etc.	[3, 149, 157, 158]
Filament-binding proteins	Tropomyosin, myosins, synapsin, spectrin, troponin, vinculin, metavinculin, actin filament-associated protein, girdin, SWAP70, synaptopodin, PDZ and LIM domain protein 4, etc.	[111, 149, 159–161]
Bundling proteins	Fascin, fimbrin, villin, erythrocyte membrane protein band 4.9, etc.	[159, 162–164]

**Table 2:** Some ABPs are involved in different immune-inflammation diseases.

ABP	Diseases involved	Effects on the inflammatory environment	Ref.
Gelsolin	Myelination and remyelination of the peripheral nervous system (PsA)	Inhibits migration of macrophages and clears macrophages Shows a negative correlation between C-reactive protein and erythrocyte sedimentation rate	[68, 70]
T*β*4	Liver injury and lung injury inflammation	Blocks the production of proinflammatory cytokines Proinflammatory response	[71, 73]
Profilin-1	Lung injury inflammation	Proinflammatory response	[73]
Filamin	Inflammatory state of melanoma cells	Inhibits TNF, IL-1, toll receptor, and TRAF2-induced NF-*κ*B activation	[74–76]
Villin-1	Gastrointestinal inflammation	Increased expression of villin-1 inhibits gastrointestinal inflammation Regulates immunogenic IEC death	[77]
SYNPO	Inflammatory bowel diseases	Regulates intestinal mucosal susceptibility and permeability	[78]
Cortactin	Inflammation in the cremaster muscle Neural inflammation	Regulates actomyosin contractility and leukocyte transendothelial migration Supports leukocyte transmigration	[79, 80]

**Table 3:** ABPs involved in different cancer diseases and their expression.

Cancer	ABPs	Correlation	Ref.
Breast cancer	ACTN4, AFAP-110, CORO1C, girdin, transgelin, ANLN, ARP2	Positive correlations	[85, 87–89, 92, 165, 166]
Gastric cancer	Fascin, fascin-1, ANLN, CAPZA1, CORO1C, YAP, ARP3	Positive correlations	[31, 94, 96–99]
Lung cancer	VASP, profilin-1, cofilin-1, profilin, fascin, ezrin, TWF1, fascin-1, ARP2	TWF1 and cofilin-1: negative correlation; VASP, profilin-1, profilin, fascin, ezrin, and fascin-1: positive correlations	[100, 102, 103, 105–107, 166]
Prostate cancer	SWAP70, PDLIM4, CORO1C, transgelin	PDLIM4: negative correlation; SWAP70, CORO1C, and transgelin: positive correlations	[111–113, 165]
Liver cancer	ANLN, 50 kDa protein	Positive correlations	[116, 117]
Pancreatic cancer	Destrin, cortactin, fascin-1	Positive correlations	[121, 122]
Esophageal cancer	Paxillin, tensin, villin-1, talin, profilin-1, profilin-2, fascin, girdin	Positive correlations	[124–128]
Melanoma	Espin, cortactin, filamin	Positive correlations	[132–135]
Colorectal cancer	Transgelin, ARP2/3, fascin	Positive correlations	[137, 140, 166]
Basal cell carcinoma, trichoblastoma, and trichoepithelioma	Drebrin	Positive correlations	[138]
